# Quality of fresh organic matter affects priming of soil organic matter and substrate utilization patterns of microbes

**DOI:** 10.1038/srep10102

**Published:** 2015-05-11

**Authors:** Hui Wang, Thomas W. Boutton, Wenhua Xu, Guoqing Hu, Ping Jiang, Edith Bai

**Affiliations:** 1 State Key Laboratory of Forest and Soil Ecology, Institute of Applied Ecology, Chinese Academy of Sciences, Shenyang, 110016, China; 2 University of Chinese Academy of Sciences, Beijing, 100049, China; 3 Department of Ecosystem Science and Management, Texas A&M University, College Station, TX 77843-2138, USA

## Abstract

Changes in biogeochemical cycles and the climate system due to human activities are expected to change the quantity and quality of plant litter inputs to soils. How changing quality of fresh organic matter (FOM) might influence the priming effect (PE) on soil organic matter (SOM) mineralization is still under debate. Here we determined the PE induced by two ^13^C-labeled FOMs with contrasting nutritional quality (leaf vs. stalk of *Zea mays* L.). Soils from two different forest types yielded consistent results: soils amended with leaf tissue switched faster from negative PE to positive PE due to greater microbial growth compared to soils amended with stalks. However, after 16 d of incubation, soils amended with stalks had a higher PE than those amended with leaf. Phospholipid fatty acid (PLFA) results suggested that microbial demand for carbon and other nutrients was one of the major determinants of the PE observed. Therefore, consideration of both microbial demands for nutrients and FOM supply simultaneously is essential to understand the underlying mechanisms of PE. Our study provided evidence that changes in FOM quality could affect microbial utilization of substrate and PE on SOM mineralization, which may exacerbate global warming problems under future climate change.

Human activities such as land use change and fossil fuel combustion are expected to directly and indirectly alter plant community structure and the quantity and quality of plant litter^1^,^2^. Previous studies have shown that leaf nitrogen (N) concentration tends to decrease and leaf lignin concentration tends to increase in response to elevated atmospheric CO_2_^3^,^4^. As fresh organic matter (FOM) inputs are delivered to the soil, its decomposition and soil organic matter (SOM) mineralization are two primary pathways of soil C cycling. Therefore, if the quality of FOM is changed, it may affect (i) FOM decomposition and soil respiration at the ecosystem scale^5^,^6^,^7^,^8^, and (ii) the intensity of priming effect (PE) on SOM mineralization^9^,^10^. A considerable amount of research has examined the first potential consequence. However, less is known about how changing quality of FOM may influence PE on SOM mineralization.

PE is defined as the changes in SOM mineralization after the inputs of exogenous substrate^9^. Our current understanding of the impacts of FOM quality on PE is still under debate. The addition of higher quality substrate (indicated by lower C:N ratio and lower lignin content) has been found to lead to a greater^11^, an equal^12^, or a lower positive PE on SOM mineralization^13^,^14^ than the addition of lower quality substrate. At present, there are two theories to explain the underlying mechanisms of positive PE under FOM addition: Theory I: FOM serves as an energy source for microorganisms to synthesize extracellular enzymes capable of degrading recalcitrant SOM, facilitating SOM mineralization, i.e. the “co-metabolism” theory^15^; and Theory II: FOM stimulates soil microbial growth and aggravates nutrients limitation, increasing the mineralization of SOM which contains higher amount of N and other nutrients than FOM, i.e. the “N-mining” theory^9^,^16^. Based on the first theory, we should expect that FOM with an optimized C:N ratio matching microbial demands and with more labile C would be the most efficient to stimulate microbial activity and positive PE^17^. The optimized C:N ratio for microbes is believed to be around 20, calculated by dividing microbial C:N ratio (10) by the C assimilation yield of microbial biomass (0.5)^18^,^19^. Hence, under natural conditions, leaf residues with a C:N ratio closer to 20 and lower lignin content would be expected to yield a larger PE than stem or root residues with a higher C:N ratio and higher lignin content. On the contrary, based on the second theory, FOM with higher C:N ratio would lead to higher N limitation and greater PE on SOM mineralization. Of course, these two mechanisms do not necessarily have to be alternative, but may happen simultaneously. However, those contradictory findings of PE induced by FOM with different quality may be because one mechanism dominates over the other in different cases^17^. Nevertheless, both theories highlight the potentially important role of soil microbial community in regulating PE on SOM mineralization. If we understand how the microbial community responds to different FOM addition while studying PE, we may be able to explain the contradiction and predict the effects of FOM quality on PE.

The quality of FOM may influence soil microbial community structure because microbes exhibit substrate preference^20^. For example, FOM with higher C:N ratio and higher lignin content tended to increase the relative abundance of fungi and actinomycetes which are adapted to nutrient poor environments^21^,^22^, whereas FOM of higher quality has been found to facilitate the growth of Gram negative bacteria^23^. Therefore, the magnitude and direction of PE may be altered by changing quality of FOM due to the modification of microbial community structure. However, our understanding is still limited by the scarce information on microbial responses to different FOM addition. Here we used ^13^C-labeled FOM to track C flow from FOM to microbial phospholipid fatty acids (PLFA) and to quantify PE on SOM mineralization simultaneously. Leaf and stalk litter from ^13^C-labelled *Zea mays* L. were used as FOM of different chemical quality based on their contrasting C:N ratio, nutrient content, and lignin content. We hypothesized that stalk (i.e. lower quality FOM) would induce higher positive PE based on the “N-mining” theory because its high C:N ratio means it would lead to more severe N limitation. The objectives of this study were to: (i) determine how the quality of FOM affects PE and which one of the above mentioned mechanism was predominant; (ii) investigate whether the quality of FOM modifies the structure and substrate utilization pattern of soil microbial community; and (iii) address the possible links between soil microorganisms and PE. Two forest soils (Larch Plantation soil and Secondary Forest soil) with different resource history and soil properties were used to test the generality of our findings.

## Results

### CO_2_ production and priming effect

**Figure 1:**
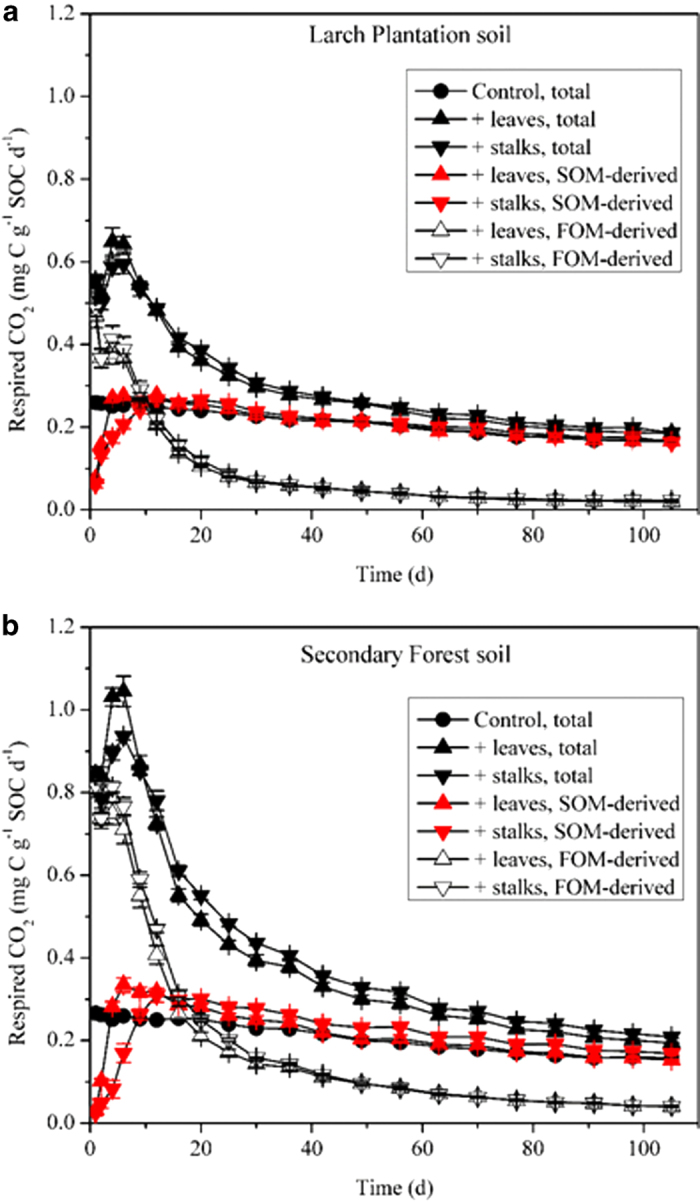
Dynamics of total, soil organic matter (SOM)-derived, and fresh organic matter (FOM)-derived CO_2_ flux (mg C g^-1^ SOC d^-1^) over the 105-day incubation for Larch Plantation soil (**a**) and Secondary Forest soil (**b**) which were treated with different FOMs. Means ± 1SD (n = 4) are shown.

For both soils, microbial respiration of FOM amended soils was significantly higher than that of control (*P* < 0.01) (Fig. 1). Temporal dynamics of CO_2_ production were similar in two soils (Fig. 1). During 0 ~ 9 d of the incubation, leaf treatment had higher total CO_2_ respiration, higher SOM-derived CO_2_, but lower FOM-derived CO_2_ than stalk treatment. During 9 ~ 30 d of the incubation, total CO_2_ respiration and FOM-derived CO_2_ were higher under stalk treatment than under leaf treatment, but SOM-derived CO_2_ was similar under both FOM treatments. During 30 ~ 105 d of the incubation, there was no difference in FOM-derived CO_2_ between the two treatments, but SOM-derived CO_2_ under stalk treatment was significantly higher than that under leaf treatment (Fig. 1, Table 1). At the end of the 105 d incubation, cumulative CO_2_ production under stalk treatment was 2.18% and 7.69% higher than under leaf treatment for Larch Plantation soil and Secondary Forest soil, respectively (Table 1). For Larch Plantation soil, cumulative SOM mineralization was not different between treatments, but for Secondary Forest soil, cumulative SOM mineralization under stalk treatment was 3.57% higher (*P* < 0.05) than that under leaf treatment (Table 1).

**Figure 2:**
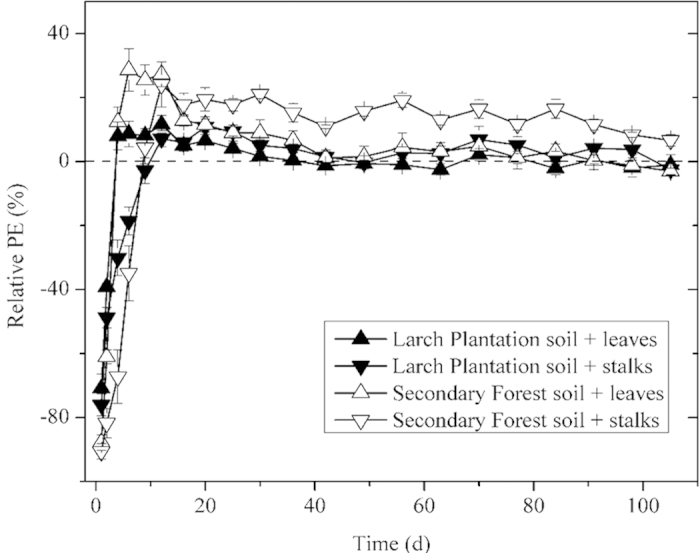
Temporal dynamics of relative priming effect (PE) for different fresh organic matter (FOM) treatments over the incubation period. Results are means ± 1SD (n = 4) on every single time-point.

**Figure 3:**
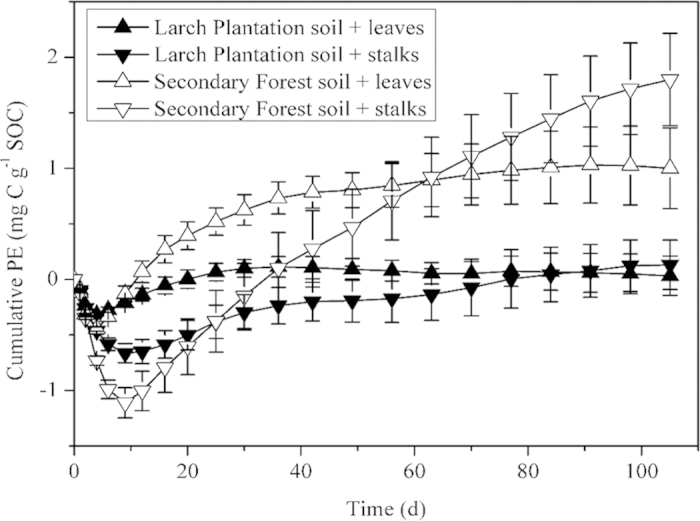
Changes of cumulative priming effect (PE) for different fresh organic matter (FOM) treatments with the incubation time. Means ± 1SD (n = 4) are shown.

Temporal dynamics of relative PE were similar for both FOM treatments and the two soil types throughout the incubation period: all started with a short phase of strong negative PE and then went into a long phase of relatively stable positive PE (Fig. 2). Stalk treatment generally had larger variations of PE than leaf treatment for both soils, with a longer negative PE (9 days for stalk treatment and 2 days for leaf treatment) in the first phase and a stronger (*P* < 0.05) positive PE in the later phase (16 ~ 105 d) (Fig. 2). The cumulative PE after 105 d incubation was positive for both treatments and both soils, and was higher in stalk treatment than in leaf treatment (Fig. 3).

### Microbial community structure and ^13^C incorporation into PLFA

**Figure 4:**
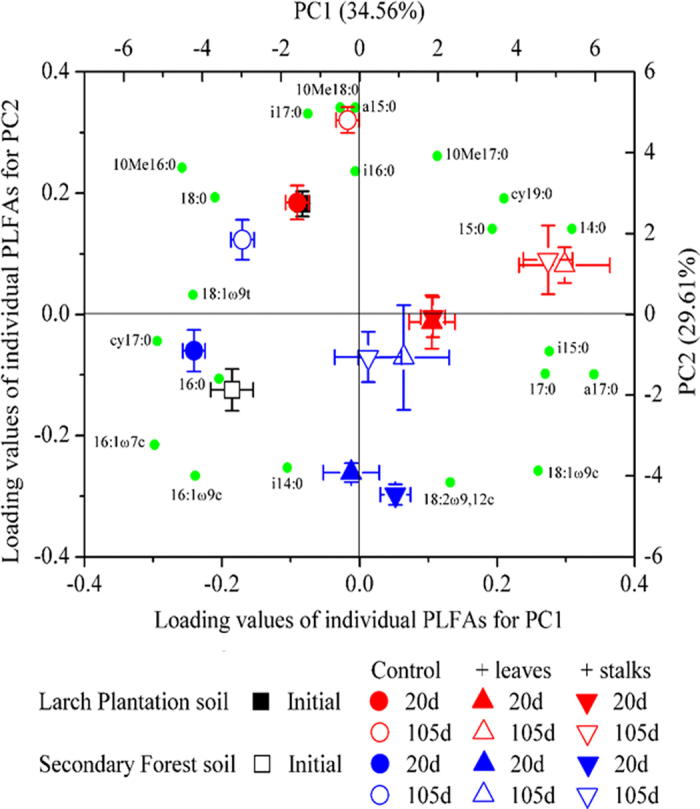
Principal components analysis (PCA) of phospholipid fatty acid (PLFA) relative abundance (%) as affected by fresh organic matter (FOM) treatments at 20 d and 105 d of the incubation for both soils. Both score plot of treatments and loading values of individual PLFAs are shown.

FOM addition significantly increased the concentration of both total PLFAs and the PLFAs associated with each microbial taxonomic grouping (*P* < 0.05) at both sampling times (Table 2). Bacteria to fungi ratio was not significantly affected by FOM addition in most cases (*P* > 0.05). The relative abundance of bacteria and fungi was enhanced (*P* < 0.05), whereas the relative abundance of actinomycetes was decreased (*P* < 0.05) by FOM addition (Table 2). However, there was no difference between the two FOM treatments (*P* > 0.05) (Table 2). PCA analysis showed soil microbial community structure was significantly affected by FOM addition, soil type, and incubation time, but was not different (*P* > 0.05) between the two FOM treatments, indicated by communities with the closest scores on the PC1 and PC2 (Fig. 4, Table S1).

**Figure 5:**
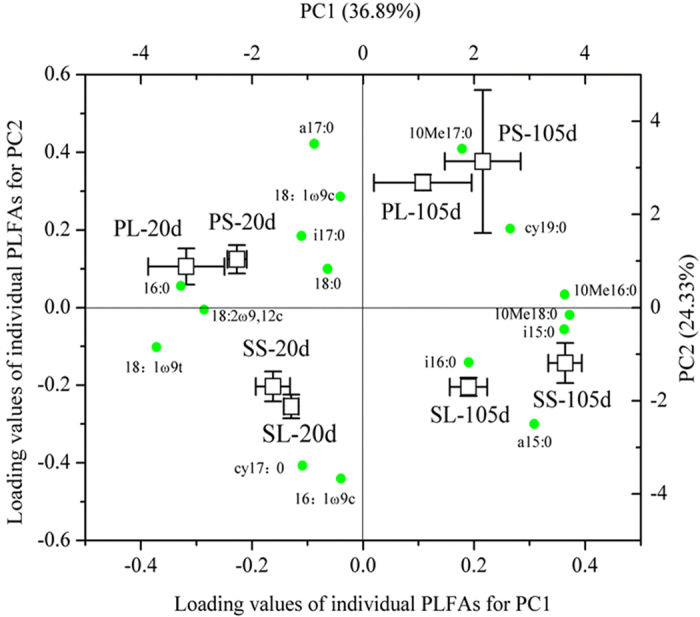
Principal components analysis (PCA) of ^13^C distribution among phospholipid fatty acids (PLFAs) (relative ^13^C incorporation into each individual PLFA (%)) as affected by fresh organic matter (FOM) treatments at 20 d and 105 d of the incubation. Both score plot of treatments and loading values of individual PLFAs are shown. PL, Larch Plantation soil+leaves; PS, Larch Plantation soil+stalks; SL, Secondary Forest soil+leaves; SS, Secondary Forest soil+stalks.

The distribution of FOM-derived ^13^C incorporation into PLFAs was strongly affected by FOM treatment, soil type, and incubation time (Fig. 5, Table S1). PC1 and PC2 explained 36.89% and 24.33% of the total variance of ^13^C distribution among PLFAs, respectively. Actinomycetes (10Me16:0; 10Me17:0; 10Me18:0) showed a strong positive loading on PC1 while fungi (18:2ω9,12c; 18:1ω9c; 18:1ω9t) showed a negative one (Fig. 5). For both sampling times, ^13^C distribution proportion within Gram-positive bacteria (Table 3) and common PLFAs (16:0 and 18:0, data not shown) under leaf treatment was higher (*P* < 0.05) than that under stalk treatment. In contrast, ^13^C distribution proportion within fungi and actinomycetes under stalk treatment tended to be higher (*P* < 0.05) than that under leaf treatment for both soils (Table 3). The highest proportion of FOM-derived C to total C was found in fungal PLFAs (Table 3). The proportion of FOM-derived C to total C in PLFAs of bacteria and fungi generally decreased with incubation time, whereas that of actinomycetes was significantly higher (*P* < 0.05) at 105 d of the incubation than at 20 d (Table 3).

## Discussion

For both soils and both treatments, FOM addition generally reduced SOM mineralization (negative PE) during the early stage of the incubation, and stimulated SOM mineralization (positive PE) after that (Fig. 1, Fig. 2). These temporal variations of PE agree with previous reports^14^,^24^. Negative PE has often been explained as the result of “preferential substrate utilization”, meaning that the preference of microorganisms for substrates switched from relatively recalcitrant SOM to amended FOM^24^,^25^. Negative PE has been found previously when highly labile substances such as glucose or sucrose was added^15^. For plant residue addition, a recent study suggested negative PE only happened when high quality grass litter was added but not when low quality bracken litter was added^14^. The discrepancy between our results and the above mentioned study may be attributed to differences in the land cover/land use history of the soils utilized in our work. The forest soils we employed in this study receive litterfall and root inputs that are relatively low quality and characterized by high C:N ratio and lignin content. When we added the higher quality, more labile FOM amendments to these soils, the microbial community preferentially consumed these amendments and caused a negative PE on SOM mineralization. After the initial short period of negative PE, positive PE began and lasted till the end of the incubation (Fig. 2). The dynamic of positive PE, i.e. an initial large pulse followed by a smaller and more prolonged flux, is consistent with most previous findings^26^,^27^. The initial pulse has been explained as effect of soluble C input leached from plant residues^27^.

It is interesting to note that high quality FOM (leaf) treatment switched from negative PE to positive PE faster than low quality FOM (stalk) did; however, its superiority only persisted for a very short period (4 ~ 12 days) and then stalk treatment showed higher PE than leaf treatment (Fig. 2). We believe this is because microbial biomass increased during the negative PE period until they had largely consumed the resources associated with the added FOM. As FOM resources became depleted, microbes had to increase utilization of SOM to meet their energy and nutrient requirements, resulting in a positive PE. For high quality FOM addition, microbial biomass grew faster, requiring less time to reach positive PE. This hypothesis can be supported by the greater microbial growth on the 20 d of the incubation under leaf treatment (Table 2). Later, PE got into a stable period and stalk treatment stimulated a greater positive relative and cumulative PE than leaf treatment did (Fig. 2, Fig. 3). As introduced above, this phenomenon is consistent with the “nutrient mining” theory (Theory II) because low quality FOM would lead to more severe nutrient limitation and thereby greater PE, but not with the “co-metabolism” theory (Theory I) which suggests that high quality FOM would yield greater PE due to more sufficient energy for the synthesis of SOM-degrading enzymes. Therefore, our results supported the dominance of “nutrients mining” mechanism during the stable positive PE period. However, for the short period when leaf treatment had higher positive PE than stalk treatment, “co-metabolism” theory may be predominant.

Based on PLFA analyses, we found that both the size and structure of the soil microbial community were significantly altered by FOM addition (Fig. 4, Table 2). During both sampling events, FOM addition stimulated the growth of total microbial biomass and every microbial group (Table 2), which is consistent with many previous findings^28^,^29^. However, we found the bacteria to fungi ratio was not changed significantly by FOM addition, suggesting relative stable microbial community structure. For the relative abundance, we found a significant increase of bacteria and fungi, whereas the relative abundance of actinomycetes declined due to FOM addition, which indicated that FOM addition was more beneficial for bacteria and fungi.

At 20 d of the incubation, PLFAs of bacteria, fungi and actinomycetes under leaf treatment were all higher than those under stalk treatment (Table 2). However, this difference did not last till the end of the incubation (Table 2). While leaf addition stimulated more growth of microbial biomass, none of the relative abundance of bacteria, fungi, and actinomycetes was different between the two FOM treatments, implying minimal effect of FOM quality on microbial composition. Nevertheless, we found that the proportions of FOM-derived C to total C in fungal and actinomycetic PLFAs were generally higher under stalk treatment than under leaf treatment at 20 d of the incubation (Table 3). In contrast, the proportion of FOM-derived C to total C in bacterial PLFAs (especially Gram positive bacteria) was much higher under leaf treatment (Table 3). These findings suggested that fungi and actinomycetes were better adapted to nutrient poor environment than bacteria, which has been reported before^14^,^30^.

Bacteria contributed more to the metabolism of fresh organic substrate as indicated by the higher ^13^C distribution proportion in bacterial PLFAs than other microbial groups under both treatments (Table 3), which is in accord with many previous findings^31^,^32^,^33^ and could mainly be attributed to the higher relative abundance of bacteria in these soils (Table 2). However, the higher proportion of FOM-derived C to total C in fungal PLFAs (Table 3) suggested that fungi was more efficient in using fresh organic substrate than bacteria and actinomycetes, which has also been reported before^34^. Gram-positive and Gram-negative bacteria did not show difference in using FOM-derived C. In contrast to the other microbial groups, actinomycetes had higher proportion of FOM-derived C to total C with increasing incubation time, indicating their competitive advantage under nutrient poor conditions after exhaustion of labile substrate.

At 20 d and 105 d of the incubation, positive PE was found to be higher under stalk treatment than under leaf treatment (Fig. 2). However, microbial biomass was found to be higher under leaf treatment than under stalk treatment at 20 d of the incubation and had no difference at 105 d of the incubation (Table 2). In addition, microbial community composition was not different between the two FOM treatments, indicated by the relative abundance of PLFA (Fig. 4, Table 2). These results together suggested that microbial biomass and microbial structure were not determinants of PE. This is contrary to the “co-metabolism” theory because if this mechanism existed, higher microbial biomass would have caused higher PE since microbes at the same time would use more SOM. Our results suggested that some FOM-stimulated microbes might use FOM only and did not necessarily use more SOM and cause positive PE.

Results of ^13^C distribution in PLFA biomarkers further suggested that fungi were responsible for using FOM but not promoting PE, while bacteria were the group responsible for positive PE. First, for bacteria, we found their biomass increased significantly in response to leaf and stalk addition (Table 2), but only a small proportion of the increased C was from ^13^C-labelled FOM (Table 3). So the increased C must be from SOM, meaning potential positive PE on SOM mineralization. The higher proportion of FOM-derived C to total C under leaf treatment was also consistent with lower PE we observed under leaf treatment (Fig. 2, Fig. 3, Table 3). Therefore, it is reasonable to speculate that bacteria are the microbial group responsible for positive PE. This viewpoint has been proposed before^26^, but some evidence did not support this claim^28^,^34^. Secondly, for fungi, we also found an increase of their biomass by FOM addition, especially by leaf treatment (Table 2). However, a high proportion of the increase was from ^13^C-labelled FOM (Table 3), meaning fungi may not be the major contributor to the increase of SOM mineralization and positive PE. Finally, for actinomycetes, their abundance was less than 10% of total microbial abundance (indicated by PLFA) in our studied soils (Table 2); hence their role in regulating PE should be minor. The average C:N ratio of bacteria, fungi, and actinomycetes is 5, 12, and 5 respectively^35^. We believe the reason for higher contribution to positive PE by bacteria is that they are more N-limited and they need N from SOM once their abundance increases. Fungi and actinomycetes have been found to acclimatize to N-limiting conditions much better than bacteria^21^,^36^. Of course, fungi and actinomycetes may also contribute to positive PE because the optimum substrate C:N ratios for them (as explained above) are still lower than C:N ratios of added FOM. These results further supported the “nutrient mining” theory, which purports that the more nutrient limited group of microbes will be responsible for positive PE.

**Figure 6:**
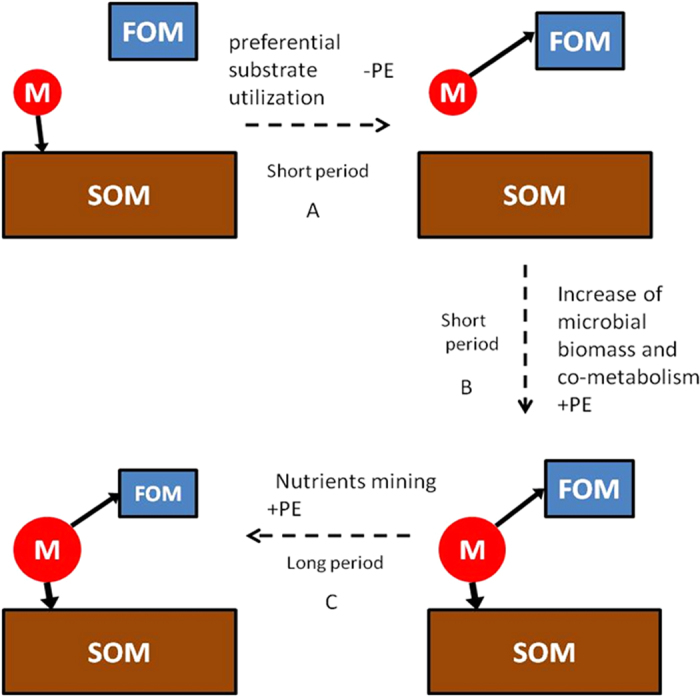
Conceptual model showing the temporal dynamics of the priming effect (PE) and its underlying mechanisms during the incubation of our studied soils. The solid arrows represent the tendency of microbes to utilize different substrates; the dotted arrows show the underlying mechanisms of priming. “M” in red circles represents soil microbes; FOM represents fresh organic matter; and SOM represents soil organic matter. They are for illustrative purposes only and do not represent actual shape or size.

In summary, we propose a conceptual model to explain the underlying mechanisms of relative PE during the incubation of our soils (Fig. 6). Addition of leaf and stalk FOM first caused negative relative PE for a short period of time (less than 10 days, A in Fig. 6) due to preferential substrate utilization, and then positive PE prevailed till the end of the incubation. It took less time for leaf treatment to switch from negative PE to positive PE than for stalk treatment because the increase of microbial biomass stimulated by FOM addition was higher under leaf treatment. With increasing microbial biomass, microbes at the same time used more SOM (co-metabolism) and caused positive PE. During this short period (B in Fig. 6), positive PE was higher under leaf treatment than under stalk treatment because microbes were mainly C limited^37^ and leaf FOM had higher labile C, stimulating higher microbial growth. Quickly after this initial boom of microbial biomass, microbes became limited by other nutrients such as N and had to use SOM for these nutrients (nutrients mining). Then positive PE reached a stable state (C in Fig. 6) and was higher under stalk treatment than under leaf treatment because the C:N ratio of stalk was higher, causing higher N limitation. Bacteria were the microbes mainly responsible for positive PE during this period (C in Fig. 6) because they were more limited by N. If future climate change reduces the quality of litter^3^,^4^, positive PE may be increased due to “microbial N mining” from SOM, causing higher SOM mineralization and CO_2_ emission fluxes and exacerbating global warming problems. It should be noted that although this conceptual model is applicable to both soils we studied, the generality of this model requires further assessment on account of the limitation of our approach: the fact that we used two parts of the same plants but not the same part of a plant grown either in “current climatic conditions (e.g. standard CO_2_ conditions” or in “future climatic conditions (e.g. increased CO_2_ conditions)”.

In conclusion, the two soils examined in this study showed consistent results: negative PE appeared in the early period after FOM addition, which suggested that soil microbes preferentially utilized more labile substrate. Soils amended with leaf tissue switched faster from negative PE to positive PE than soils amended with stalk tissue, due to greater microbial growth stimulated by leaf FOM during this short period. After the exhaustion of easily available nutrients, positive PE was higher under stalk treatment than under leaf treatment from approximately 16 d until the end of the incubation. During this period of stable positive PE, microbial biomass and microbial community structure did not seem to be the determinants of PE. Instead, microbial C:N ratios and microbial demand for carbon and other nutrients were major determinants of PE. If more carbon was needed, most microbes preferentially used FOM with more labile C, which would not induce positive PE; and if more N was needed, they had to use SOM with higher N content, which would cause positive PE. Therefore, our results suggested that FOM quality and microbial demands acted together to determine PE. Future studies should consider incorporating this effect of litter quality on PE into C cycling models for better understanding and predicting ecosystem responses to climate change.

## Methods

### Study area and soils

Soils were collected from the 0–15 cm depth increment in a 60-year-old natural secondary forest, and a 35- to 45-year-old larch plantation located at the Qingyuan Experimental Station, Liaoning Province, China (41°51′ N, 124°54′ E, 500 ~ 1100 m above sea level). For detailed descriptions of the Secondary Forest and Larch Plantation sites, see Yang et al.^38^. Soils at both sites are typical brown forest soils with a silty loam texture and are classified as Udalfs according to the second edition of US Soil Taxonomy^38^. The chemical and physical properties of the soils are shown in Table 4. At each site, six soil cores (15 cm × 15 cm, 0-15 cm) were randomly collected after removal of surface plant litter and then bulked together. Fresh soils were passed through a 2 mm sieve and visible plant residues were removed prior to storage at 4 °C for later measurements.

### Production of ^13^C-labeled plant material

*Zea mays* L. (yellow field maize, Paymaster Hybrid 8951) was grown under field conditions at the USDA/ARS Rice Research Unit in Beaumont, Texas. A portion of one row of corn at the grain-filling portion of the life cycle was covered with a transparent chamber (3 m × 0.9 m × 2.4 m, L × W × H) constructed with PVC tubing and polyethylene film, and then labeled with 50 L of 99.3 atom % ^13^CO_2_ delivered continuously between 1200-1600 hours, after which the chamber was removed. Maize plants continued to grow in the field until they were harvested 28 days post-labeling. Leaves and stalks were separated from the whole plant, dried at 50 °C, and then finely milled (passed through a 2 mm screen) prior to incubations. Stalks had higher C:N ratio and higher lignin content than leaves (Table 5), and were therefore considered as lower quality FOM.

### Incubation design

For the incubation, six treatments (2 soil types×3 FOM treatments) with 8 replicates were set up: Larch Plantation soil control (without FOM addition), Larch Plantation soil+leaves, Larch Plantation soil+stalks; Secondary Forest soil control, Secondary Forest soil+leaves, Secondary Forest soil+stalks. Four replicates were used to measure soil CO_2_ efflux and δ^13^CO_2_ (n = 4); the other four replicates were prepared for destructive sampling at 20 d of the incubation to quantify soil microbial community structure. We checked the respiration rate in the flasks used for destructive sampling and those used for PE during the initial 3 times of air sampling (i.e. 1 d, 2 d and 4 d after FOM addition), and found there was no difference (*P* > 0.05) between them. The rates of C added as FOM to each of the two soil types were chosen to mimic rates of annual C input (litterfall+fine root) into top 0-15 cm soils under field conditions (see Table 4)^39^: calculated 0.691 g C kg^−1^ dry soil for Larch Plantation soils and 1.905 g C kg^−1^ dry soil for Secondary Forest soils for our incubation.

First, 200 g fresh soil at ambient field moisture content was added to a 1.0 L Mason jar. The jar was sealed with Parafilm^®^ M (Bemis Company, Neenah, WI) during incubation to minimize evaporation without affecting gas exchange. Soils were pre-incubated for 10 days at 20 °C to stabilize the disturbance of previous soil preparation and to recover microbial activity that may have been diminished during the storage period. Then, FOM was added to pre-incubated soils and mixed to distribute the material homogeneously. This procedure was also conducted in controls although no FOM was added. These jars were then incubated for 105 days (post FOM addition) at 20 °C in darkness. Soil water content was maintained at original soil moisture throughout the incubation by daily addition of deionized water as necessary.

### Soil CO_2_ efflux and δ^13^CO_2_

Four replicates of each treatment were utilized to measure soil CO_2_ efflux and δ^13^CO_2_ for the entire duration of the incubation experiment. The accumulation method was used to quantify CO_2_ production^40^. Each jar was first flushed with CO_2_-free air for 1 h to reduce headspace CO_2_ concentration to < 10 ppm, then hermetically sealed for 6 h with a silicone stopper fitted with two three-way stopcocks (the highest CO_2_ concentration was about 2800 ppm after air accumulation, thus the environment in the jar would not be anoxic). After that, 150 ml gas sample was taken from each jar with a syringe through the three-way stopcock and injected into an evacuated aluminum foil airbag (200 ml). A gas substitution procedure was conducted to avoid gas exchange during sampling: 150 ml CO_2_-free air was injected to the jar using the syringe; the syringe was gently pumped up and down for 5 times; and then gas samples were taken. The decrease of CO_2_ concentration due to gas substitution was corrected before statistical analysis. CO_2_ concentrations were determined at 1, 2, 4, 6, 9, 12, 16, 20, 25, 30, 36, 42, 49, 56, 63, 70, 77, 84, 91, 98 and 105 day after FOM addition. Gas samples were analyzed for CO_2_ concentration and δ^13^CO_2_ values within 24 h after sampling using a carbon dioxide isotope analyzer (CCIA-36d-EP; LGR, Mountain View, CA, USA). The reproducibility and accuracy of the analytical procedure checked with reference gas (361.5 ppm, with a δ^13^C value of -8.935) were better than 1.0 ppm and 0.4 ppm for CO_2_ concentration, whereas 0.4%o and 0.2%o for δ^13^CO_2_, respectively (n = 4).

### Soil sampling and chemical analysis

Before treatments and after pre-incubation, four samples of each soil type were collected to measure microbial biomass C (MBC), NO_3_^−^-N and NH_4_^+^-N concentrations. In addition, four subsamples of each treatment were sampled at 20 d and 105 d after FOM addition; these soil samples were freeze-dried and ground with a ball mill (Retsch MM200; Haan, Germany) for analysis of phosopholipid fatty acids (PLFAs).

The concentration of C and N in initial bulk soil and FOM were determined by an element analyzer (Model CN, vario Macro Elementar, GmbH, Germany). Klason-lignin content of FOMs was measured according to the gravimetric method of Theander and Westerlund^41^. The δ^13^C signatures of SOC and FOM were determined using a stable isotope ratio mass spectrometer (Thermo Finnigan, DELTA Plus XP) interfaced with an elemental analyzer (Flash EA 1112). MBC was determined by fumigation-extraction method^42^.

### PLFA analyses

Soil samples before treatments (after pre-incubation) and at 20 d and 105 d after treatments were analyzed for PLFA using a modified Bligh-Dyer method^28^. Briefly, 5 g of freeze-dried soil was extracted with phosphate buffer-chloroform-methanol (0.8:1:2), and then the phospholipids were separated and eluted with methanol on a silicic acid column. PLFAs were mildly derivatized to fatty acid methyl esters (FAMEs) with alkaline methanol. Methyl nonadecanoate (19:0, Sigma-Aldrich, St. Louis, MO, USA) was used as the internal standard. The concentrations of FAMEs were identified using a Thermo Finnigan Trace GC-MS System, and the ^13^C values of FAMEs were determined using a Thermo Scientific Trace GC Ultra attached to a Finnigan MAT 253 IRMS, as described by Rubino et al^31^.

For the identification of soil microbial community composition, the following PLFA designations were used: Gram-positive bacteria i14:0, a15:0, i15:0, i16:0, a17:0, i17:0; Gram-negative bacteria 17:0cy, 19:0cy, 16:1ω7c, 16:1ω9c; actinomycetes 10Me16:0, 10Me17:0, 10Me18:0; and fungi 18:1ω9c, 18:1ω9t and 18:2ω9,12 c. Short or odd-chain saturated PLFAs (14:0, 15:0, 17:0) were considered non-specific bacterial markers. The term “bacteria” in this paper refers to Gram-positive bacteria, Gram-negative bacteria and also these non-specific bacteria collectively. Common saturated PLFAs (16:0, 18:0) were not assigned to a taxonomic group but were considered as additional measures of total microbial biomass^33^,^43^,^44^. Soil microbial community structure was investigated using relative PLFA abundance (mol%).

Among the total 21 PLFAs identified, only 16 of those were present in sufficient amount for accurate isotopic analysis. Hence, 5 PLFAs without sufficient amount (14:0, 15:0, 17:0, i14:0 and 16:1ω7c) were not included in subsequent calculations. The proportion of FOM-derived C to total C in each PLFA (*P*_*i*_) was determined using the following equation^31^:


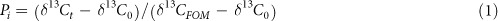


where δ^13^C_t_ and δ^13^C_0_ are the δ^13^C values (%o) of individual PLFA in the “FOM+soil” treatments and control, respectively; δ^13^C_FOM_ is the δ^13^C (%o) of the labeled FOM.

The proportion of FOM-derived C to total C in each microbial group (e.g. bacteria, fungi) was calculated as:


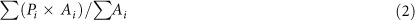


^13^C distribution proportion in each microbial group (e.g. bacteria, fungi) was calculated as:


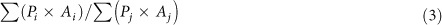


where *P*_*i*_ and *A*_*i*_ are the proportion of FOM-derived C to total C and the abundance (accounting for molecular C content) of each PLFA for a special microbial group (e.g. bacteria, fungi), respectively; while *P*_*j*_ and *A*_*j*_ (*j* = 1,2,∙∙∙∙∙∙16) are those for all 16 PLFAs.

### Calculations

To calculate the contribution of added FOM to CO_2_ respiration, a two end-member mixing model was used:


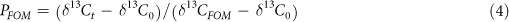


where *P*_FOM_ is the proportion of FOM-derived CO_2_; δ^13^C_t_ and δ^13^C_0_ are the δ^13^C values (%o) of respired CO_2_ in the “FOM+soil” treatments and control, respectively; δ^13^C_FOM_ is the δ^13^C value (%o) of FOM.

PE induced by the addition of FOM was calculated by comparing the amount of SOC-derived CO_2_ under FOM treatments with the amount of CO_2_ under control^15^, according to the following equation:


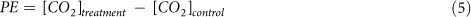



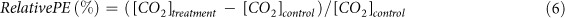


where, [CO_2_]_treatment_ and [CO_2_]_control_ represent SOM-derived CO_2_ efflux (or cumulative CO_2_ respiration) in the “FOM+soil” treatments and control, respectively.

Cumulative CO_2_ production, cumulative SOM mineralization, cumulative FOM mineralization, and cumulative PE for a specific time span could be estimated by integrating CO_2_ efflux, SOM-derived CO_2_ efflux, FOM-derived CO_2_ efflux, and absolute PE over time respectively.

### Statistical analyses

Statistical analyses were carried out using the SPSS 16.0 package (SPSS, Chicago, IL, USA). Homogeneity of variances of data was tested prior to analyses and data were log-transformed when necessary (*P* < 0.05). Repeated measures analysis of variance (ANOVA) were used to examine differences in CO_2_ respiration (including total CO_2_, SOM-derived CO_2_ and FOM-derived CO_2_) and PE (including relative PE and cumulative PE) with time among FOM treatments. One-way ANOVA or Independent-Samples T Test was used to analyze the effects of FOM treatments on the cumulative CO_2_ respiration for a specific time span, PLFA abundance, proportion of FOM-derived C in each PLFA groups and ^13^C incorporation into each PLFA groups for each sampling time and each soil respectively. Impacts of treatments on microbial community structure (PLFA relative abundances) and ^13^C distribution among PLFAs (relative FOM-derived ^13^C incorporation into each individual PLFA) were assessed by principal components analysis (PCA). Two-way ANOVA was used to test the effects of FOM quality, sampling time, and their interactions on the first component (PC1) and the second component (PC2) of PCA. An α value of 0.05 was chosen to indicate statistical significance.

## Author Contributions

H.W., W.H.X. and E.B. conceived and designed the experiments. H.W., G.Q.H., T.W.B., and P.J. performed the experiments. H.W., G.Q.H. and E.B. analyzed the data. H.W., T.W.B. and E.B. wrote the manuscript; other authors provided editorial advice.

## Additional Information

**How to cite this article**: Wang, H. *et al.* Quality of fresh organic matter affects priming of soil organic matter and substrate utilization patterns of microbes. *Sci. Rep.* **5**, 10102; doi: 10.1038/srep10102 (2015).

## Figures and Tables

**Table 1:** Cumulative CO_2_ production, soil organic matter (SOM) mineralization and fresh organic matter (FOM) mineralization during 0~9 d, 9~30 d, 30~105 d, and the whole period of the incubation.

	**Larch Plantation soil**		**Secondary Forest soil**	
	**+leaves**	**+stalks**	**+leaves**	**+stalks**
CO_2_ production (mg C kg^-1^ soil)				
0~9 d	171.13a	161.78b	367.46a	338.38b
9~30 d	266.91b	277.65a	510.62b	557.11a
30~105 d	560.89b	581.29a	895.06b	971.00a
0~105 d	998.93b	1020.72a	1773.14b	1866.49a
0~9 d	0.21a	0.16b	0.22a	0.12b
9~30 d	0.53a	0.54a	0.59a	0.61a
30~105 d	1.44b	1.49a	1.43b	1.59a
0~105 d	2.18a	2.19a	2.24b	2.32a

FOM mineralization (% of added FOM)				
0~9 d	14.86a	15.70a	14.15b	14.93a
9~30 d	13.01b	14.29a	12.87b	14.82a
30~105 d	12.37a	12.95a	13.38a	13.67a
0~105 d	40.25a	42.93a	40.40b	43.43a

Different letters in the same line denote significant differences (*P* < 0.05) between two FOM treatments based on Independent-Samples T Test (n = 4).

**Table 2:** Concentration (nmol g^-1^ soil) and relative abundance (%) of selected phospholipid fatty acid (PLFA) groups in fresh organic matter (FOM) treated soils on 0 d, 20 d, and 105 d of the incubation.

	**Larch Plantation soil**														**Secondary Forest soil**								
	**Total PLFAs**	**G+**			**G-**	**Bacteria**				**Fungi**		**Actinomycetes**			**Total PLFAs**		**G+**		**G-**	**Bacteria**	**Fungi**	**Actinomycetes**	
**PLFA concentration (nmol g**^**-1**^ **soil)**																							
***Initial***	74.76	25.17	15.38				42.47			13.74			7.74		104.00		32.50	22.78		57.72	19.81	9.98	
***20d***																							
Control	63.61c	21.04c	13.06c					35.69c		11.67c			6.34c		94.10c		30.34c	20.63c		52.99c	17.23c		9.02c
+leaves	119.19a	40.69a	24.37a					68.35a		22.95a			10.49a		181.30a		60.45a	37.06a		102.14a	36.27a		14.43a
+stalks	101.26b	34.32b	21.91b					58.91b		19.53b			8.61b		161.35b		54.30b	33.51b		92.04b	31.84b		12.26b
***105d***																							
Control	67.14b	22.87b		13.40b				38.14b		11.38b			7.02b		96.35b		32.68b	18.94b		54.02b	16.34b		10.04b
+leaves	102.52a	37.55a		19.39a				60.28a		18.60a			8.74a		141.05a		50.18a	27.41a		81.53a	25.34a		11.85a
+stalks	104.95a	37.22a		20.72a				61.22a		19.15a			9.57a		147.67a		51.75a	28.68a		84.37a	26.60a		13.01a

**PLFA relative abundance (%)**																							
***Initial***	—	33.65			20.58				56.80	18.39			10.34		—		31.26	21.92		55.52	19.05		9.59
***20d***																							
Control	—	33.07a			20.54a				56.11b		18.35b			9.97a		—	32.26b	21.93a		56.34a	18.30b		9.59a
+leaves	—	34.15a			20.44a				57.34a		19.25a			8.80b		—	33.34a	20.44b		56.34a	20.00a		7.96b
+stalks	—	33.90a			21.64a				58.18a		19.29a			8.50b		—	33.66a	20.77b		57.05a	19.73a		7.60b
***105d***																							
Control	—	34.06b			19.94a				56.79b		16.97b			10.46a		—	33.92c	19.65a		56.07c	16.96b		10.42a
+leaves	—	36.64a			18.92b				58.81a		18.14a			8.52b		—	35.58a	19.44a		57.82a	19.97a		8.39b
+stalks	—	35.46a			19.75a				58.33a		18.26a			9.11b		—	35.04b	19.42a		57.14b	18.01a		8.81b

Letters in the same column for each sampling time denote significant differences (*p* < 0.05) between FOM treatments (n = 4). -, not available.

**Table 3:** Proportion of fresh organic matter (FOM) derived C to total C and ^13^C distribution among selected phospholipid fatty acid (PLFA) groups in FOM treated soils on 0 d, 20 d, and 105 d of the incubation.

	**Larch Plantation soil**					**Secondary Forest soil**									
	**G+**	**G-**	**Bacteria**	**Fungi**	**Actinomycetes**	**G+**		**G-**		**Bacteria**		**Fungi**		**Actinomycetes**	
**Proportion of FOM-derived C to total C in PLFAs (%)**															
***20d***															
+leaves	5.37a	4.96a	5.22a	9.66b	1.80b	9.67a		10.70a		10.03a		15.69a		5.87a	
+stalks	4.78b	4.77a	4.78b	10.40a	2.55a	7.68b		9.70b		8.39b		16.32a		6.82a	
***105d***															
+leaves	4.49a	3.81a	4.27a	6.62a	4.08a		9.17a		9.02a		9.12a		12.13a		7.66b
+stalks	4.27a	3.74a	4.09a	7.64a	4.96a		9.40a		9.18a		9.33a		12.38a		9.27a

^**13**^C distribution among PLFA groups (%)*															
***20d***															
+leaves	25.39a	12.67b	38.06a	26.96b	2.29b		25.12a		15.09a		40.20a		26.19b		3.91b
+stalks	23.94a	13.84a	37.78a	30.97a	3.35a		22.69b		15.66a		38.36b		30.26a		4.86a
***105d***															
+leaves	29.64a	12.06a	41.70a	22.63b	6.51a		30.36a		14.68a		45.05a		21.78a		6.39b
+stalks	27.18b	12.34a	39.52a	26.20a	8.57a		30.61a		14.73a		45.34a		22.22a		8.12a

Letters in the same column for each sampling time denote significant differences (*P* < 0.05) between FOM treatments (n = 4). *Note:* The proportion of FOM-derived C to total C in each microbial group (e.g. bacteria, fungi) was calculated as: 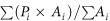; ^13^C distribution proportion in each microbial group (e.g. bacteria, fungi) was calculated as: 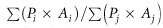; where *P*_*i*_ and *A*_*i*_ are the proportion of FOM-derived C to total C and the abundance of each PLFA for a special microbial group (e.g. bacteria, fungi), respectively; while *P*_*j*_ and *A*_*j*_ (*j* = 1, 2∙∙∙∙∙∙16) are those for all 16 PLFAs detected.

^*^As the results of common saturated PLFAs (16:0, 18:0), which were not assigned to a taxonomic group (bacteria, fungi or actinomycetes), were not shown, the sum of these % was less than 100%.

**Table 4:** Chemical and physical characteristics of soils. Mean values are shown (n = 4).

	**Larch Plantation soil**	**Secondary Forest soil**
SOC (%)	3.31	4.48
TN (%)	0.31	0.38
C:N	10.67	11.69
δ^13^C_V-PDB_ (%o)	-26.61	-27.22
pH (H_2_O)	5.5	5.8
SWC (%, dry basis)	42.4	50.5

Water-stable aggregate size fraction (%)		
Macroaggregate (250 ~ 2000 μm)	16.21	32.54
Microaggregate (53 ~ 250 μm)	37.70	37.99
Silt plus clay particles (<53 μm)	46.09	29.47
Forest C input (litterfall+fine root) (g m^-2^ y^-1^)*	229.4	594.3
Basal respiration (mg C kg^-1^ soil d^-1^)	8.03	11.04
SOC-specific basal respiration (mg C kg^-1^ SOC d^-1^)	242.6	246.5
Microbial biomass (mg C kg^-1^ soil)	529.6	727.9
NO_3_^-^-N (mg kg^-1^ soil)	28.03	29.81
NH_4_^+^-N (mg kg^-1^ soil)	7.25	9.12

SOC, soil organic carbon; TN, total nitrogen; SWC, soil water content.

^*^Forest C input was reported by Yang et al. (2010) ^ref.^ 39.

**Table 5:** Chemical characteristics of fresh organic matter (FOM) used in soil incubations.

	**C (%)**	**N (%)**	**C:N**	**Klason-lignin (%)**	**Ash (%)**	**δ**^**13**^**C**_**V-PDB**_ (%o)
Stalks	44.8	1.0	44.8	11.4	4.0	132.6
Leaves	43.0	1.5	29.4	8.6	9.6	142.9
